# LegalEye: Multimodal Court Deception Detection Across Multiple Languages

**DOI:** 10.3390/bs15121707

**Published:** 2025-12-09

**Authors:** Rommel Isaac A. Baldivas, Nivedha Sreenivasan, So Young Kang, Alexandra My-Linh Miller, Megan Chacko, Shreya Krishnan, Carmen Ayala, Esperanza Ayala, Dohyeong Kim

**Affiliations:** 1 Department of Computer Science, The University of Texas at Dallas, Richardson, TX 75080, USA; rommelisaac.baldivas@utdallas.edu (R.I.A.B.); nivedha.sreenivasan@utdallas.edu (N.S.); alex.miller2@utdallas.edu (A.M.-L.M.); megan.chacko@utdallas.edu (M.C.); shreya.krishnan@utdallas.edu (S.K.); carmen.ayala@utdallas.edu (C.A.); esperanza.ayala178@gmail.com (E.A.); 2 Department of Police Science, Konkuk University, Chungju 27478, Republic of Korea; 3 School of Economic, Political and Policy Sciences, The University of Texas at Dallas, Richardson, TX 75080, USA; dohyeong.kim@utdallas.edu

**Keywords:** deception detection, multimodal, machine learning, cross-linguistic modeling

## Abstract

This study introduces LegalEye, a multimodal machine-learning model developed to detect deception in courtroom settings across three languages: English, Spanish, and Tagalog. The research investigates whether integrating audio, visual, and textual data can enhance deception detection accuracy and reduce bias in diverse legal contexts. LegalEye uses neural networks and late fusion techniques to analyze multimodal courtroom testimony data. The dataset was carefully constructed with balanced representation across racial groups (White, Black, Hispanic, Asian) and genders, with attention to minimizing implicit bias. Performance was evaluated using accuracy and AUC across individual and combined modalities. The model achieved high deception detection rates—97% for English, 85% for Spanish, and 86% for Tagalog. Late fusion of modalities outperformed single-modality models, with visual features being most influential for English and Tagalog, while Spanish showed stronger audio and textual performance. The Tagalog audio model underperformed due to frequent code-switching. Dataset balancing helped mitigate demographic bias, though Asian representation remained limited. LegalEye shows strong potential for language-adaptive and culturally sensitive deception detection, offering a robust tool for pre-trial interviews and legal analysis. While not suited for real-time courtroom decisions, its objective insights can support legal counsel and promote fairer judicial outcomes. Future work should expand linguistic and demographic coverage.

## 1. Introduction

Perjury in court poses a serious threat to the fairness and credibility of the judicial system. False testimony can distort legal outcomes, increasing the risk of wrongful convictions and undermining public trust in justice (Crank & Curtis, 2023). The ability to detect perjury accurately is therefore essential to ensuring equitable trials. However, the legal system continues to rely heavily on judges and jurors to assess witness credibility—a process limited by subjective judgment and the inherent fallibility of human intuition (Denault & Dunbar, 2019). Despite efforts from legal and psychological researchers, findings suggest that perjury often goes undetected, and courts lack standardized criteria for identifying deceptive testimony. An analysis of 230 court rulings, for instance, found that perjury was rarely identified, highlighting a systemic shortfall in detection practices (Krukovsky & Mosechkin, 2024). Deception detection begins long before trial, during police interrogations, which shape the evidence admitted in court. Yet, research shows that even trained officers struggle to identify deception reliably—often performing at or below chance levels (Yarbrough, 2020). In courtroom settings, the responsibility shifts to jurors and attorneys, who typically lack formal training in deception detection. This gap in expertise further weakens the system’s ability to evaluate testimony effectively (Vrij & Mann, 2001).

Human communication includes both verbal and non-verbal elements. While non-verbal cues are often assumed to reveal deception, empirical evidence shows that even professionals are poor judges of these signals (Brennen & Magnussen, 2023). Efforts to create standardized frameworks for interpreting non-verbal behavior have not only proven ineffective but also risk producing false positives. Consequently, greater emphasis has been placed on verbal cues, with criminologists developing structured guidelines for evaluating spoken and written statements. Still, these verbal analysis methods frequently misclassify truthful accounts as deceptive, reflecting a troubling decline in diagnostic accuracy (Hauch et al., 2016). Despite their limitations, non-verbal cues remain a critical component of deception analysis (Yarbrough, 2020).

Recent advances in technology have opened new possibilities for improving perjury detection. The growing availability of multimodal data—such as video, audio, and transcripts from courtroom proceedings—has enabled the development of artificial intelligence (AI) and machine learning approaches to analyze deception. For example, Andrewartha introduced a linguistic analysis model to identify deceptive language (Andrewartha, 2008), while Minzner explored machine learning tools to assess testimonial credibility (Minzner, 2008). More recently, Crank and Curtis proposed a multimodal AI system that analyzes facial expressions, speech, and text simultaneously, offering promising improvements in accuracy (Crank & Curtis, 2023).

Building on this momentum, researchers at the University of Michigan developed a neural network-based model for multimodal deception detection using datasets comprising visual, auditory, and textual inputs (Sen et al., 2022). Their findings showed that neural networks, particularly when combined using Late Fusion techniques, outperformed traditional classifiers such as Random Forests. However, these models have been tested primarily on English-language data within the U.S. legal context. To broaden applicability, our work seeks to expand these models to multilingual, multicultural courtroom settings while addressing potential gender and racial biases. LegalEye serves as the first multimodal deception detection model evaluated across English, Spanish, and Tagalog with race- and gender-balanced datasets for all three languages—ultimately moving toward more equitable and globally relevant perjury detection systems.

## 2. Background

Deception detection has long since been a primary component of the legal process.

This section explores traditional methods for deception detection, including manual analysis of subjects (Frank & Ekman, 2004; Porter et al., 2002) and the use of neuroscience methods (Ford, 2006; Schauer, 2009). Additionally, we explore emerging AI methods for identifying deception (Crank & Curtis, 2023; Perez-Rosas et al., 2015; Wu et al., 2018; Zuckerman et al., 1981) and the algorithms that preceded them (Andrewartha, 2008).

### 2.1. Traditional Methods for Deception Detection

Jurors and judges often assess a witness’s credibility based on non-verbal cues and the consistency of testimony; however, this method has significant limitations in objectively identifying perjury (Fisher, 1997). Research shows that jurors’ evaluations are frequently influenced by biases related to the defendant’s race, gender, or social status (Minzner, 2008), compromising the fairness of proceedings. Such biases suggest that credibility assessments are shaped more by subjective perceptions than by objective standards, undermining the integrity of the trial process (Rand, 2000). The credibility of police testimony also raises serious concerns. Officers may offer incomplete or distorted accounts in court, a phenomenon often referred to as “testilying,” where police manipulate testimony to secure convictions (Cunningham, 1999; Slobogin, 1996). Additionally, while attorneys are legally obligated to prevent perjury, some may tolerate or even facilitate it. Studies indicate that lawyers are sometimes aware of perjury but fail to intervene, further eroding the reliability of courtroom testimony (McKoski, 2012; Silver, 1994).

Early research explored the potential of non-verbal cues to detect deception. Frank and Ekman (2004) analyzed facial expressions and body movements during testimony, identifying micro-expressions potentially linked to lying (Frank & Ekman, 2004). Similarly, Porter et al. (2002) found that vocal patterns—such as pitch variation and hesitancy—were associated with deceptive behavior (Porter et al., 2002). While these studies suggest a role for non-verbal indicators in perjury detection, their reliability is limited by individual differences and external factors like stress, which can influence behavior regardless of truthfulness.

More recent approaches have examined perjury through neuroscientific methods. Schauer (2009) used fMRI to observe increased activity in the frontal and temporal lobes during acts of deception (Schauer, 2009), while Ford (2006) employed EEG to measure cognitive load during lying, noting distinct changes in brainwave patterns (Ford, 2006). Although these methods offer quantitative insights into deceptive behavior, practical barriers—including high costs, legal admissibility, and ethical concerns—restrict their courtroom application. Polygraph tests and psychological analysis techniques have also been used, but their reliance on stress responses rather than deception itself limits their validity as reliable evidence (Andrewartha, 2008). These findings highlight a growing need for more objective, scientifically grounded tools. Ultimately, effective perjury detection requires moving beyond intuition and toward a systematic, evidence-based approach that addresses the limitations of traditional methods.

### 2.2. Machine Learning for Deception Detection

In recent years, artificial intelligence (AI) and machine learning (ML) have gained increasing attention in the field of perjury detection. These technologies offer promising tools for analyzing courtroom behavior, particularly through multimodal data sources that integrate verbal and non-verbal cues. Andrewartha (2008) developed an early linguistic-based algorithm to detect perjury by identifying standardized deceptive expressions in courtroom testimony (Andrewartha, 2008). Similarly, Minzner (2008) applied machine learning models to assess testimony credibility (Minzner, 2008), while Crank and Curtis (2023) introduced a more advanced multimodal AI system that simultaneously analyzes speech, text, and facial expressions (Crank & Curtis, 2023). Their model demonstrated improved accuracy in identifying deceptive behavior and highlighted the potential for AI-driven systems to be more effectively integrated into legal settings.

Multimodal AI-driven systems have the potential to analyze the four factors defined by Zuckerman et al. (1981), namely attempted control, which can lead to dissonance between various leaky channels. Furthermore, the principle that arousal arises from deception, and that truth-telling and lying trigger different autonomous responses, lends credence to the use of automated detection systems. Numerous theories, such as the conditioned response theory, the conflict theory, and the punishment theory, seek to explain the cause of autonomous responses, however explaining their source is outside of this paper’s scope. Rather, we focus on identifying patterns of autonomous behavior across deceptive and truthful testimonies and analyzing emotions in alignment with the affective approach, which posits that deception gives rise to emotional displays resulting from suppressed feelings rather than arousal. Additionally, we study length of pauses in speech in accordance with the theory of cognitive factors in deception, which states that increased cognitive complexity of telling lies may lead to longer pauses in speech and increased hesitation.

Building on these foundations, Perez-Rosas et al. (2015) created a multimodal deception detection tool using a dataset of 121 real-life English courtroom videos (Perez-Rosas et al., 2015). The system analyzes textual data via a bag-of-words approach and encodes unigrams and bigrams, while visual features—such as facial expressions and hand gestures—are manually annotated using the MUMIN coding scheme. Employing Decision Trees and Random Forests, the model achieves 60–75% accuracy, outperforming human annotators who scored below 60% even when given access to full video, audio-only, or silent footage. Wu et al. (2018) expanded upon this dataset to develop a model incorporating visual, audio, and textual modalities (Wu et al., 2018). They used GloVe embeddings for textual input to preserve semantic structure, and Mel-frequency Cepstral Coefficients (MFCCs) for audio, which were compiled into an “audio dictionary.” Visual inputs focused on micro-expressions and motion-based features. By applying late fusion to combine outputs from the three modalities, they found that Support Vector Machines (SVM) and Random Forests achieved the highest accuracy, with the visual modality alone reaching over 80%—significantly outperforming human judgment.

While these models show promise, ethical and practical concerns remain. Suchotzki and Gamer (2024) emphasize the risks associated with AI in legal contexts, particularly regarding algorithmic bias, privacy violations, and overreliance on automated systems (Suchotzki & Gamer, 2024). They call for caution and ethical oversight in applying AI to high-stakes decisions like perjury detection. Prome et al. (2024) provide a systematic review of machine learning and deep learning approaches to deception detection (Prome et al., 2024). Their study systematically categorizes existing algorithms and datasets, highlighting key strengths, limitations, and unresolved research challenges. Notably, Gogate et al. (2023) introduce a deep learning-based multimodal fusion framework that integrates textual, vocal, and visual inputs, achieving higher accuracy than unimodal approaches (Gogate et al., 2023). Their results emphasize the effectiveness of multimodal fusion in capturing the nuanced and context-sensitive characteristics of deceptive behavior. Taken together, these findings indicate that AI-driven perjury detection offers significant promise, particularly when leveraging diverse data modalities. Nonetheless, translating these advancements into real-world courtroom applications will require not only continued technical refinement but also careful attention to ethical, legal, and procedural concerns.

## 3. Materials and Methods

LegalEye builds upon prior work published by the University of Michigan (Sen et al., 2022), Perez-Rosas et al. (2015) and Wu et al. (2018). In this section, we document our dataset expansion and analysis process, including our standards for rigor and quality of videos. We provide an overview of our dataset distribution and additionally elaborate on the feature extraction process for textual, audio, and visual modalities. We also detail how cross-validation is performed.

### 3.1. Framework

A team from the University of Michigan pioneered the use of neural networks for multimodal deception detection, analyzing visual, audio, and textual data from an English-language dataset of 121 courtroom videos (Sen et al., 2022)—the same dataset used by Perez-Rosas et al. (2015) and Wu et al. (2018). They compared Random Forest and Neural Network classifiers across individual modalities and found that Neural Networks combined with Late Fusion achieved the best results. Building on this approach, our model—implemented within the “LegalEye” framework—expands the scope of deception detection by incorporating data from Spanish and Tagalog court proceedings, along with a broader representation of racial and gender diversity. This enriched dataset not only diversifies the existing data from the University of Michigan but also enhances global applicability and minimizes bias in legal settings.

**Figure 1:**
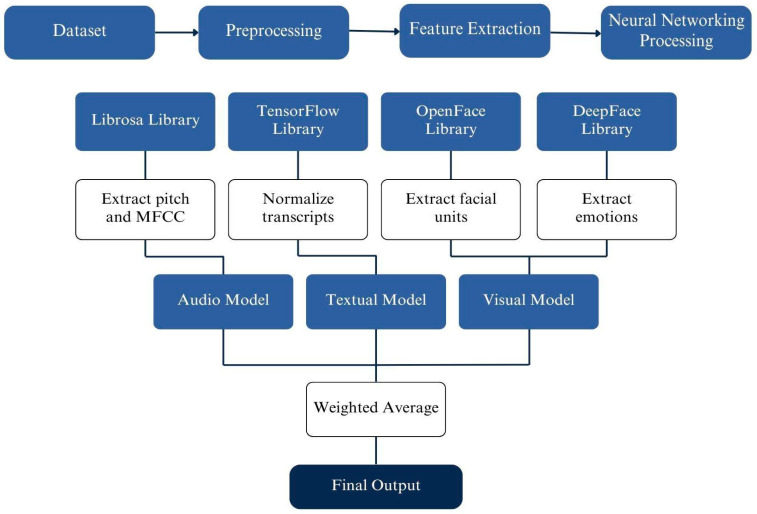
An overview of LegalEye’s framework and each modality’s structure.

**Figure 2:**
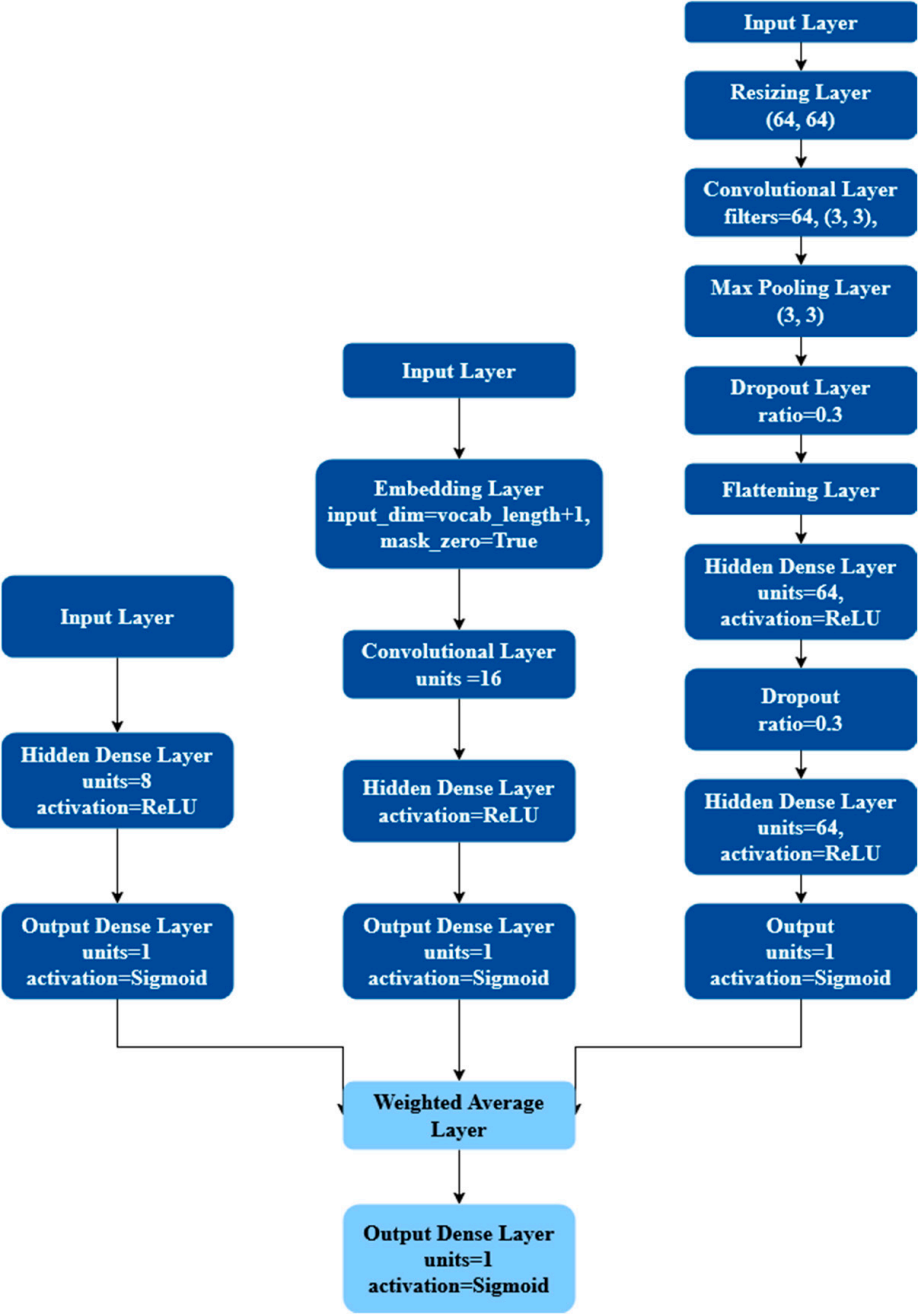
LegalEye’s late fusion model architecture. Dark blue shapes represent a part of an individual modality and light blue shapes represent parts of the Late Fusion model.

LegalEye is a multimodal analysis tool for detecting deception that processes textual, audio, and visual inputs. We manually collected and transcribed court videos in Tagalog and Spanish, extracted features from each modality, and applied Neural Networks to identify patterns indicative of deceptive behavior. Our decision to adopt Neural Networks combined with Late Fusion was inspired by the successful outcomes achieved by the University of Michigan team. The refined framework of our approach, illustrated in Figure 1, demonstrates how integrating diverse linguistic data with advanced machine learning techniques can improve the accuracy of perjury detection. Figure 2 details how we used Neural Networks to create a Late Fusion model. This integration of extended datasets and improved analytical methods represents a significant advancement in creating more robust and unbiased legal analysis tools.

### 3.2. Data Collection and Processing

Due to the legal setting from which all data is taken, we consider all scenarios to be high stakes, meaning the deceiver is highly motivated to succeed in their deception, and the listener is highly motivated to detect the deception (Ekman & Friesen, 1974). Additionally, we concern ourselves with both factual and emotional deceptions but do not differentiate between them, as emotional deception can occur alongside factual deception. As Zuckerman et al. explain, a deceiver feeling guilt must hide both their emotion and its cause to succeed in their deception (Zuckerman et al., 1981).

To train our model, we compiled datasets in English, Spanish, and Tagalog—three languages that use Romanized scripts, allowing consistency in textual feature training. Native speakers on our team transcribed the videos, enabling accurate and cost-effective data preparation. Each video focuses on the subject’s face, figure, and voice to enhance training accuracy. All videos were sourced from YouTube, featuring public courtroom trials and interviews. While many clips were taken from trial broadcasts and courtroom footage, not all were originally aired before the verdict was public. Post-verdict media framing may influence video quality or context; however, the risk of bias is minimized as our dataset focuses on subjects’ own spoken statements rather than third party commentary or framing. Many of the subjects were the defendants themselves; however, in some cases, the subject was the defendant’s prosecutor or a witness during the trial. While most subjects were documented, some could not be identified due to lack of available information. Our English dataset includes 255 subjects: 199 videos curated by our team and 56 condensed from the University of Michigan’s “Real-life Deception” dataset (originally 121 videos, reduced by merging clips featuring the same subject). To mitigate racial bias, we ensured representation of Black, White, Hispanic, and Asian individuals from across North America. The Spanish dataset comprises 109 subjects of Hispanic descent, drawn from court cases and interviews across Spain and South America. The Tagalog dataset includes 112 Filipino subjects from court proceedings and interviews in the Philippines.

The average clip durations were 25.29 s for English, 23.47 s for Spanish, and 22.27 s for Tagalog, with ranges of 8–48 s, 2–27 s, and 1–27 s, respectively. The clips in both the RealLifeDeception2016 dataset and those manually recorded by our team are labeled at the clip level based on verdicts or corroborating evidence. While a clip may contain a mix of truthful and deceptive statements, features are averaged across the entire segment, consistent with prior research. This approach reflects the realistic setting where deception is often embedded within otherwise truthful narratives.

All datasets are balanced by gender, and the Spanish dataset includes a transgender subject, Marilyn Bernasconi, allowing exploration of gender-related deception cues. We applied strict selection criteria, focusing on courtroom testimonies and interviews where the subject’s truthfulness could be verified based on case verdicts. Subjects include both defendants and witnesses from criminal and civil cases. Notable cases represented in the dataset include the trials of Jodi Arias, Trayvon Martin, and O.J. Simpson. By diversifying languages, ethnicities, and courtroom contexts, our dataset improves upon existing deception detection resources and supports more robust, bias-aware model training (Oravec, 2022).

To enhance model accuracy, our team took deliberate steps to limit extraneous data in the training videos. During recording, we cropped footage to focus solely on the primary subject, reducing background distractions and minimizing the number of faces detected by OpenCV (version 4.9.0.80). This streamlined the facial expression analysis process, as fewer subjects improved data labeling efficiency (King & Neal, 2024). We also ensured that only the subject’s voice was captured, removing background speakers to isolate vocal features for audio analysis. Video cropping was done by the same individuals who sourced the clips, so they were aware whether each clip was truthful or deceptive. Furthermore, these steps did not involve subjective judgment, interpretation, or labeling decisions, so prior knowledge could not influence the data itself. Thus, the risk of bias in the dataset creation process is minimal.

As shown in Table 1, our English dataset improves upon prior efforts by offering greater racial balance. Unlike the University of Michigan dataset, we included an approximately equal number of Black, White, and Hispanic subjects—about 40 videos per group—to reduce racial bias. However, only 13 videos feature Asian subjects, reflecting a broader issue: Asian Americans, despite representing 5.6% of the U.S. population, constitute just 1.5% of the federal prison population (Hu & Esthappan, 2017). This underrepresentation is mirrored in our dataset, potentially limiting model generalizability across racial groups.

Ground truth labels for “truthful” and “deceptive” were assigned based on the correlation between a subject’s testimony and the verdict of their case. Each clip was linked to a specific statement and checked against the established case outcome. If the subject was exonerated or found guilty, we assumed the verdict to be accurate. If the subject was speaking on an unrelated topic following the allegation of a crime, we consider this statement relevant to detecting deception, as the interpersonal theory (Burgoon) posits that deceivers must not only conceal facts but also emotions. Videos falling into this designation were classified as deceptive if the speaker was found guilty, or truthful if the speaker was found innocent, based on the principle that concealing guilt and holding ‘normal’ conversation is a form of emotional deception. It is important to state that our team did not subjectively judge whether subjects were being deceptive or not. Rather, labels were derived from publicly documented case outcomes. This verdict-based approach reflects a legal outcome rather than a verified truth indicator. We focused solely on the subjects’ statements, rather than other forms of evidence, such as DNA, due to limitations in the Spanish and Tagalog datasets. Unlike the U.S. judicial system, there are no public databases with consolidated trial records and outcomes for Spanish or Tagalog court systems. This made it significantly more difficult to find data for the Spanish and Tagalog datasets. Although a stricter evidence rubric would improve data quality, it would inadvertently result in a smaller dataset and additional model complexity. This procedure ensured that labeling reflected the honesty of the statement within context, rather than assuming that every utterance in a guilty case was false. Research shows that approximately 6% of criminal convictions leading to imprisonment are later overturned due to errors or wrongful judgments (Loeffler & Ridgeway, 2019). While we acknowledge this limitation, our project scope does not allow independent verification of each verdict. The ground truth distribution for each language dataset is shown in Table 2.

To extract textual features, our team manually transcribed all videos. The English dataset was transcribed by four researchers; the Spanish and Tagalog datasets were completed by native speakers. Due to limited staffing, transcripts were not cross-validated and may contain occasional transcription or translation errors. Consistency was maintained by using ellipses for pauses and unintelligible speech, and by retaining filler words (e.g., “um,” “uh”). Existing transcripts from the “Real-life Deception” dataset were reformatted to match our style. The training videos are sourced from publicly available YouTube content and have been organized on this page (accessed on 1 December 2025): https://docs.google.com/spreadsheets/d/1Q09nuzN3zPfJ7rrWQUBCS5ePL4Qcc2czVgd3cwhmoCI/edit?usp=sharing.

### 3.3. Feature Extraction

For each sub model—textual, visual, and audio—we process its input data to extract relevant features for training. Due to the unique nature of each modality, we use a specialized feature extraction process for each sub model. The feature extraction processes are detailed below, along with the model architecture for each modality.

#### 3.3.1. Linguistic Features

To examine the relationship between language and deception, our model analyzes the frequency of words used in truthful versus deceptive testimonies. Burgoon and Buller (2015) compare a series of experiments and find that deceptive responses are less likely to use self-references and group-references than truthful ones, and that deceptive responses were less clear, relevant, and personalized (Burgoon & Buller, 2015). When a deceiver is equivocating—giving a vague, ambiguous response with the purpose of deceiving their audience—they are linguistically immediate, using words that convey timelessness and spontaneity without a focus on duration and build-up. LegalEye’s textual analysis is based on the principle that certain words correlate to deception.

Using a bag-of-words approach and vocabulary frequency, LegalEye identifies linguistic patterns by quantifying how often certain words appear in each subject’s transcript. This method mirrors techniques used in criminology, where experts analyze verbal cues to assess truthfulness (Brennen & Magnussen, 2023). Unlike rigid human guidelines, LegalEye dynamically learns associations between word use and deception, adapting to patterns across the dataset. English and Spanish share many linguistic features that facilitate cross-language analysis. Both languages follow a subject–verb–object (SVO) sentence structure and have high lexical overlap due to their Latin roots. Rivera (2019) reports that 30–40% of English words have Spanish cognates, with about 90% retaining the same meaning (Rivera, 2019). While English typically follows an article–adjective–noun order, Spanish uses article–noun–adjective, maintaining semantic equivalence. Agirre et al. (2015) use semantic textual similarity (STS) to measure equivalence across English and Spanish text snippets, confirming that the two languages are often semantically aligned (Agirre et al., 2015). In contrast, Tagalog presents significant challenges. Pascasio (1961) finds notable structural differences in adjective–noun constructions and comparative forms between English and Tagalog (Pascasio, 1961). Unlike the relatively flexible syntax of Spanish, Tagalog enforces stricter word order and relies on affixes for tense, aspect, and negation (Osoblivaia, 2023). Tagalog lacks gendered grammar and shares fewer syntactic features with English and Spanish, despite historical influence from both. Schachter and Reid (2018) note that Spanish and English colonization had minimal effect on core Tagalog syntax, making standardized processing across all three languages more difficult (Schachter & Reid, 2018).

**Figure 3:**
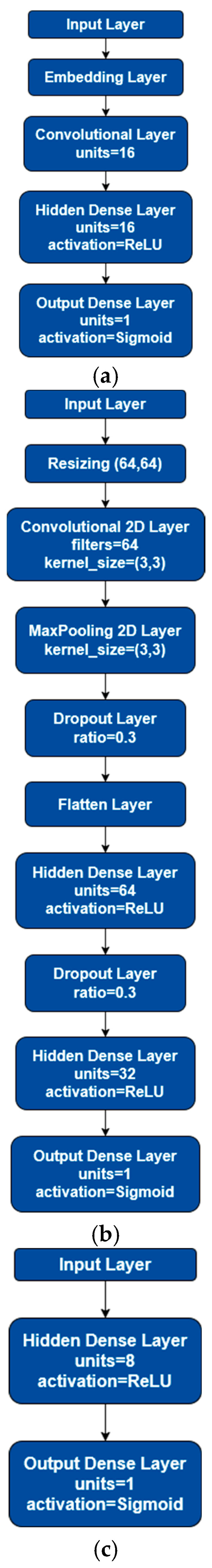
In-depth Neural Network Architectures for each modality. (**a**) CNN architecture for LegalEye’s textual modality. (**b**) CNN architecture for LegalEye’s audio modality. (**c**) NN architecture for LegalEye’s visual modality.

Despite these linguistic differences, LegalEye applies a uniform preprocessing method to maintain consistency. Pauses, inaudible speech, or video cuts are marked with ellipses (“…”), and filler sounds are standardized as “uh” (for vowel-ending fillers) or “um” (for consonant-ending fillers). Transcripts are lowercased, and punctuation is removed to normalize the input. Though TensorFlow cannot store diacritics, this limitation does not impact our model’s function, as LegalEye analyzes word frequency rather than meaning. Characters with diacritics are stored consistently and treated identically for training purposes. We used TensorFlow to convert transcriptions into datasets, which were then fed into a convolutional neural network (CNN). LegalEye uses CNNs to detect correlations between specific words and deception, under the assumption that deceptive and truthful testimonies have distinct linguistic patterns. For instance, deceptive speech may include more hesitation markers or distancing language, which the model learns over time. The CNN consists of an input layer, an embedding layer, a convolutional layer, global average pooling layer, a hidden dense layer, a dropout layer, and an output dense layer, described in Figure 3a. For all three languages, the embedding dimension, CNN units, and dense units are 16. Additionally, the hidden dense layer uses the ReLU activation function, and the output dense layer uses the Sigmoid activation function. Although RNNs are usually used for text classification tasks, Banerjee et al. (2019) explains how CNNs can also be used for classification with fixed-length word representations such as the bag-of-words model (Banerjee et al., 2019). We saw that CNNs were better suited for our use case and gave better results whereas RNNs required more technical computing power with non-impressive results.

Although we tested sequential embedding to incorporate the context and order of words, it reduced model accuracy in our case. This method attempts to derive meaning from sentence structure, which may be beneficial in larger datasets but proved unreliable given the size of ours. The bag-of-words method, which focuses on individual word presence and frequency, outperformed sequential embedding for our use case. In summary, LegalEye effectively processes multilingual textual data for deception detection by leveraging linguistic similarities between English and Spanish while accounting for structural differences in Tagalog. Using a consistent transcription method, CNNs, and frequency-based word analysis, our model offers a dynamic and scalable approach to detecting verbal deception across diverse courtroom contexts.

#### 3.3.2. Visual Features

**Figure 4:**
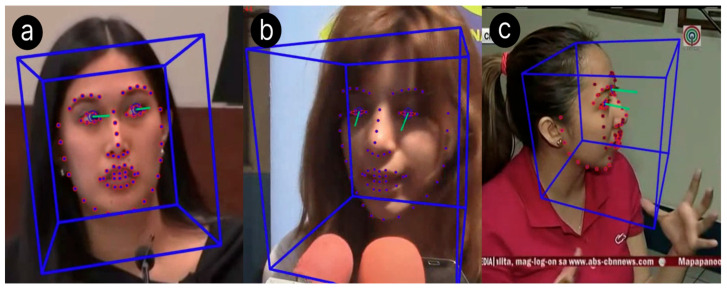
Sample screenshots showing facial displays and hand gestures from real-life trial clips. (**a**) Deceptive English trial with a side gaze (Gaze Side). (**b**) Truthful Spanish trial with a down gaze (Gaze Down). (**c**) Deceptive Tagalog trial with one hand movement (Single hand).

**Figure 5:**
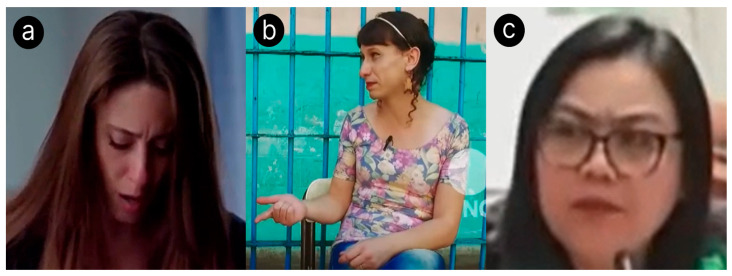
Sample screenshots showing emotions from real-life trial clips. The colored overlay on faces in each subfigure show the face that OpenFace is detecting. (**a**) Deceptive English trial exhibiting sadness. (**b**) Truthful Spanish trial exhibiting neutral. (**c**) Deceptive Tagalog trial exhibiting surprise.

The leakage hypothesis states that the face is a primary source of nonverbal deceptive cues, leading us to analyze facial expressions to identify deception (Ekman & Friesen, 1969). The principle of cognitive factors in deception, one of the four factors posited by Zuckerman et al. (1981), states that crafting lies requires increased concentration, which leads to markers of deception (Zuckerman et al., 1981). Visually, this includes increased pupil dilation and fewer illustrators—including fewer hand gestures and body movements. We choose not to consider “micro expressions”—fleeting facial movements that reveal hidden emotions (Oravec, 2022)—due to their rarity; in a study by Porter and ten Brinke (2008), only 21.95% of participants exhibit a micro expression in 2% of all expressions (Porter & ten Brinke, 2008). Furthermore, Su and Levine (2016) find that analysis of micro expressions alone is not much better than random change at identifying deception (Su & Levine, 2016). LegalEye leverages computer vision to analyze these visual signals, enhancing its ability to assess the credibility of a subject’s claims. To build our visual dataset, we extracted facial features from each video using two libraries: OpenFace (version 2.2.0) and DeepFace (version 0.0.93). OpenFace, a facial behavior analysis tool, identifies the presence and intensity of 18 facial muscle movements—known as action units—which are widely used to classify expressions (Sen et al., 2022). DeepFace, a facial attribute analysis framework, detects five core emotions: happiness, sadness, fear, surprise, and neutral. As shown in Figure 4, these emotions strongly correlate with deceptive behavior and were therefore included to improve model accuracy. Both OpenFace and DeepFace features were computed for each video frame, averaged across the entire video, and merged into a single set of visual features per clip. We chose to extract each frame from the videos for use with OpenFace and DeepFace because we found that OpenFace and DeepFace worked faster on images than videos. These libraries generated input vectors for the visual model that are 1D, with 39 features represented. Due to the lack of reliable annotators, we opted for automatic feature extraction over the semi-automatic methods used in prior research. Since the 1D input vectors for the visual features from OpenFace and DeepFace were not as complex opposed to the other two modalities, using a simple neural network, highlighted in Figure 3c, was sufficient in our use case. It was important for us to use a simpler neural network, architectural-wise, because it allowed us to train faster. Processing thousands of video frames with OpenFace and DeepFace required a vast amount of computing power. That was rewarded with high-quality results that allowed us to use a simpler model. The sample snapshots are shown in Figure 4 and Figure 5.

#### 3.3.3. Acoustic Features

Zuckerman et al. (1981) argue that the voice is a leaky channel, and that the tone of voice is more difficult for a deceiver to control than their facial expression (Zuckerman et al., 1981). This leads us to study the average of a speaker’s vocal pitch. Burgoon and Buller (2015) also find that deceptive responses show a greater pitch variety than truthful ones. Audio data, in its raw form, is represented as a signal—capturing the variation of sound waves over time (Kim & Choi, 2013). While audio signals contain a wide array of extractable features, LegalEye focuses specifically on features most relevant to deception detection: pitch and Mel-Frequency Cepstral Coefficients (MFCCs). These were selected based on prior success in deception classification studies (Sen et al., 2022; Wu et al., 2018). Pitch, also known as the fundamental frequency, is one of the most important features for detecting vocal patterns across languages. The fundamental frequency is the lowest partial in a sound wave and often serves as a reference point for other harmonics. Since human vocal pitch typically ranges between 0 and 5000 Hz (Tiwari, 2010), it offers valuable insight into both speaker identity and vocal variability under emotional or deceptive conditions. Pitch patterns often shift under stress or cognitive load, which are common during deceptive speech. MFCCs are a standard audio feature used in speech and speaker recognition. They approximate how humans perceive the spectral envelope of sounds and help distinguish subtle phonetic and prosodic differences across languages. While spectrograms and mel-spectrograms are also commonly used, our experiments confirmed that MFCCs consistently produced higher model accuracy and training stability when classifying deception. Additionally, MFCCs are sensitive to the articulation and rhythm changes that often accompany deceptive speech (Ittichaichareon et al., 2012).

**Figure 6:**
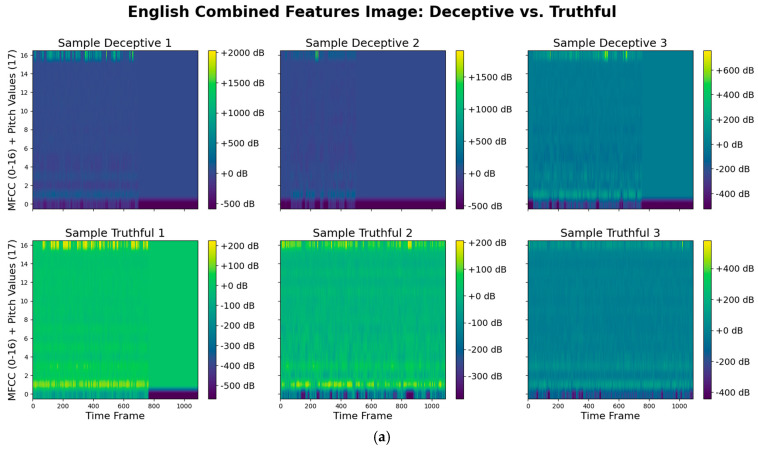

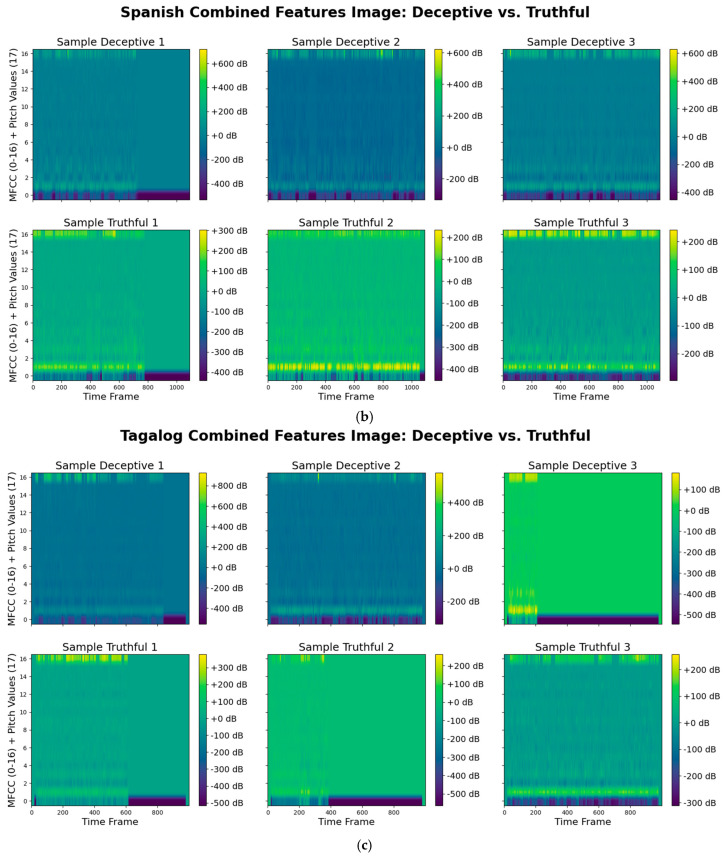
Combined Features for Deceptive vs. Truthful Sound by Language: (**a**) English, (**b**) Spanish, (**c**) Tagalog, illustrating pattern differences across truthful and deceptive speech.

For preprocessing, all video files across the English, Spanish, and Tagalog datasets were first converted into audio. The recordings originally featured stereo channels with sampling rates of 44,100 Hz or 48,000 Hz. These were resampled to a mono channel at 22,050 Hz, a frequency that provided a balance between computational efficiency and feature fidelity. Audio feature extraction was conducted using Librosa (version 0.10.1), a widely adopted Pythonlibrary (version 3.12) for music and audio analysis. Librosa’s pYIN algorithm was used to extract pitch, while its MFCC extraction function generated 2D spectral representations. A key technical challenge was the difference in dimensionality between pitch and MFCC features when these features had to be combined as input to the model. Pitch values are stored as 1D arrays across time windows, whereas MFCCs are 16D matrices representing frequency over time (16 coefficients). To resolve this mismatch and enable integrated learning, we concatenated the pitch vertically to the MFCC, essentially adding another row. This allowed us to feed the combined audio features into a Convolutional Neural Network (CNN), which excelled in capturing spatial relationships between audio cues. Figure 6 displays how the combined features would visually look as an image. While we experimented with late fusion models—which analyze pitch and MFCCs independently before merging predictions—the CNN model, with layered audio features, consistently delivered better classification accuracy and robustness across all datasets. Highlighted in Figure 3b, our CNN model used a resizing layer as another preprocessing step before the convolutional step which is flattened for the neural network stage. The convolutional step involves a 2D convolutional layer for the audio features represented as images, a 2D max pooling layer and a dropout layer. We used a smaller kernel size (3 × 3) for the convolutional layer because of the finer features in the combined feature image seen in Figure 4. The final step includes two hidden dense layers with a dropout layer between and finally an output dense layer.

### 3.4. Neural Network Model

Neural networks are a type of artificial intelligence modeled after the human brain. They consist of interconnected nodes, or “neurons,” organized in layers. Each node processes input using mathematical functions and passes the result to the next layer, enabling the network to recognize patterns and make predictions. Training a neural network involves feeding it large amounts of data and refining its internal weights through a process called backpropagation. This process compares the network’s output with known results and adjusts parameters to minimize error. Over time, the model improves its accuracy, even with new or unseen data.

A key challenge during training is overfitting, where the model becomes too tailored to the training data and fails to generalize. To reduce this, a technique called dropout is used, which randomly disables a portion of nodes during training. This encourages the model to learn broader trends rather than memorizing specific details. We used dropout throughout all the models. We also utilized early stopping in the fusion models (with a patience of 5 units) as another technique to prevent overfitting.

Furthermore, on the parameters of our models, the number of epochs—full passes through the training data—also affects learning. Because video, audio, and text data vary in complexity, LegalEye tunes the number of epochs for each modality to optimize performance. For all our models we utilized Adam optimization and binary cross-entropy as our loss function. Neural networks are widely used in speech recognition, image analysis, and medical diagnostics. Their ability to process complex, multimodal data makes them well-suited for LegalEye, which integrates visual, vocal, and linguistic features to assess deception. By leveraging this architecture, LegalEye can analyze patterns across different data types to make more accurate and informed predictions.

### 3.5. Cross-Validation

To further enhance generalization, justify the performance of our models, and reduce the risk of overfitting, we implemented *k*-fold cross-validation during training. Rather than the traditional fixed train—validation split in the datasets, each dataset is partitioned into *k* equally sized folds. For each iteration, *k* − 1 folds are used for training, and the remaining fold is the validation set. This is done *k* times so that each sample is used both in the training and validation sets. To attune to the different sizes of each language dataset, we compared training at different number of folds and decided the standard number of folds is at *k* = 5 since it had greater balance between variance during training, as well as having a sufficient size for the testing set. At this number of folds, the Spanish dataset, the smallest set, had 22 videos for the testing set, and any higher fold would only lower the size. The metrics that were tracked for evaluation were balanced accuracy and AUC. Balanced accuracy is a metric that averages the specificity and the sensitivity of the model evaluation. This was used to handle the imbalance of sample sizes between Deceptive and Truthful, especially in the Spanish dataset. Area Under the ROC Curve (AUC) was another metric used to measure the performance of the models correctly classifying in binary classification problems. This allows for a metric suited for the more balanced datasets such as the English and Tagalog datasets. The results reported in Section 4 for the individual models are the highest mean metrics in a general dataset split (75% for the training and 25% for the testing set) and cross-validation at *k* = 5 folds. For the combined models, the results report the highest mean metrics at a cross-validation with *k* = 5 folds.

## 4. Results

In this section, we analyze the individual results and the late fusion results of each sub model for all three languages—English, Spanish, and Tagalog—and provide insight on the best-performing sub model for each language. We discuss how the various sub models impact the combined model’s performance and how weight distributions affect the late fusion results, then evaluate the effectiveness of combining two of the three modalities.

### 4.1. English Model: Individual Modality

The textual model achieved a 65.08% accuracy on the English dataset—the lowest among the three languages. Despite having the largest dataset, the English model struggled due to the high variance among subjects, who came from diverse backgrounds to minimize implicit bias. Even with cross-validation, the highest mean balanced accuracy it could reach was 62.6%, performing lower than the other two languages. This diversity likely introduced inconsistent speech patterns, making it harder for the model to learn generalized deception cues. To avoid overfitting on a relatively small dataset, we limited training to 10 epochs, as additional epochs resulted in lowered training accuracy and validation accuracy. Larger datasets benefit from more epochs, as they provide greater variety for pattern learning and validation.

**Figure 7:**
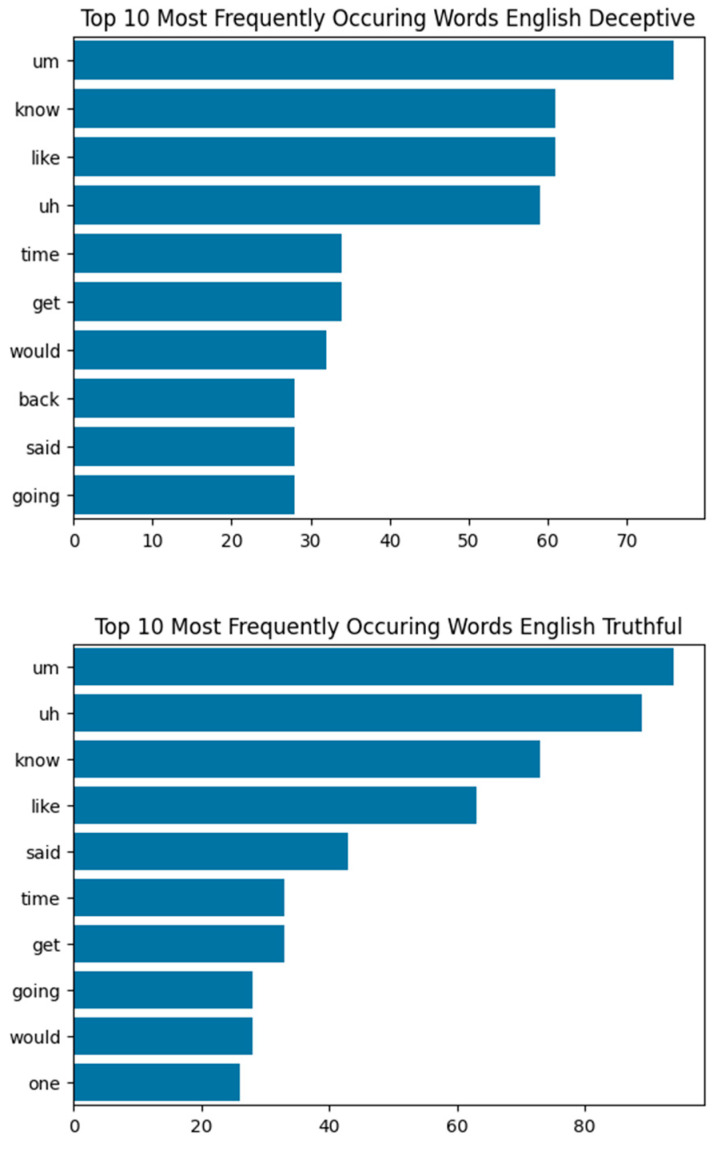
Top 10 Most Frequently Occurring Words in English Data: Deceptive vs. Truthful, demonstrating lexical similarities and differences within the data. From most to least frequent Deceptive: um (filler), know, like, uh (filler), time, get, would, back, said, going. From most to least frequent Truthful: um (filler), uh (filler), know, like, said, time, get, going, would, one.

As shown in Figure 7, common words in both deceptive and truthful English testimonies often overlap. Fillers such as “um” and “uh” are prevalent across both categories, and high-frequency words like “know,” “like,” “time,” “said,” and “going” appear in both deceptive and truthful speech. This lexical overlap makes it challenging for the textual model to distinguish deception based on word frequency alone. In contrast, the visual model achieved a remarkable 96.88% accuracy—the highest among all visual models across the three languages. The visual model on the English dataset performed extremely well with a mean balanced accuracy of 98.64% after cross-validation. Its success can be attributed to the dataset’s size and diversity, which helped the model learn visual deception cues effectively. This suggests that, when video footage is available, the visual model alone may be sufficient for reliable deception detection in English-language trials.

The audio model ranked second, with 68.75% accuracy. With cross-validation, the audio model on the English dataset reached a mean balanced accuracy of 79.94%. However, the average pitch values between deceptive (162.78 Hz) and truthful (163.32 Hz) speech were nearly identical, making differentiation difficult. Compared to Spanish and Tagalog, English showed the smallest pitch variation. Despite this, the model achieved an AUC above 70%, indicating it performed better than random guessing. While pitch alone offers limited predictive power in English, the audio model still contributes meaningful information when combined with other modalities.

### 4.2. Spanish Model: Individual Modality

The Spanish textual model achieved the highest accuracy among all languages at 81.48%, despite the dataset being smaller than the English one. This result is surprising, as fewer transcripts generally limit the variety of linguistic patterns the model can learn. One possible factor is the consistency in transcription—unlike the English dataset, which involved five scribes, the Spanish transcripts were produced by only two. This likely improved uniformity in word usage and sentence structure, enhancing the model’s learning. During cross-validation, Spanish performed with a mean balanced accuracy of 69.01%. Although the dataset is smaller, the evaluation supports that Spanish is better performing at Textual than the other two languages. Additionally, while Spanish and English share cognates and syntactic similarities (Rivera, 2019), Spanish transcripts contained more consistently structured language. Unlike the Tagalog dataset, which featured significant code-switching between English and Tagalog, the Spanish dataset used almost exclusively Spanish words. This consistency allowed the model to more easily identify patterns in the text.

**Figure 8:**
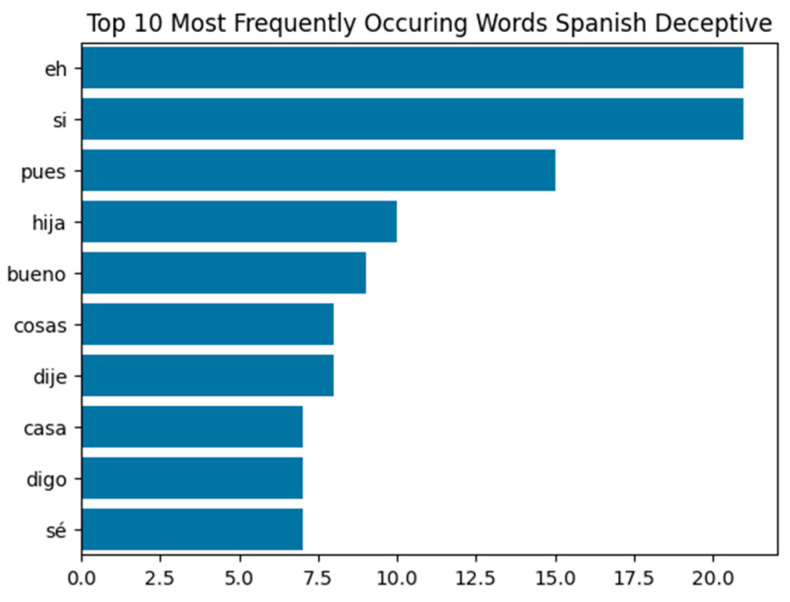

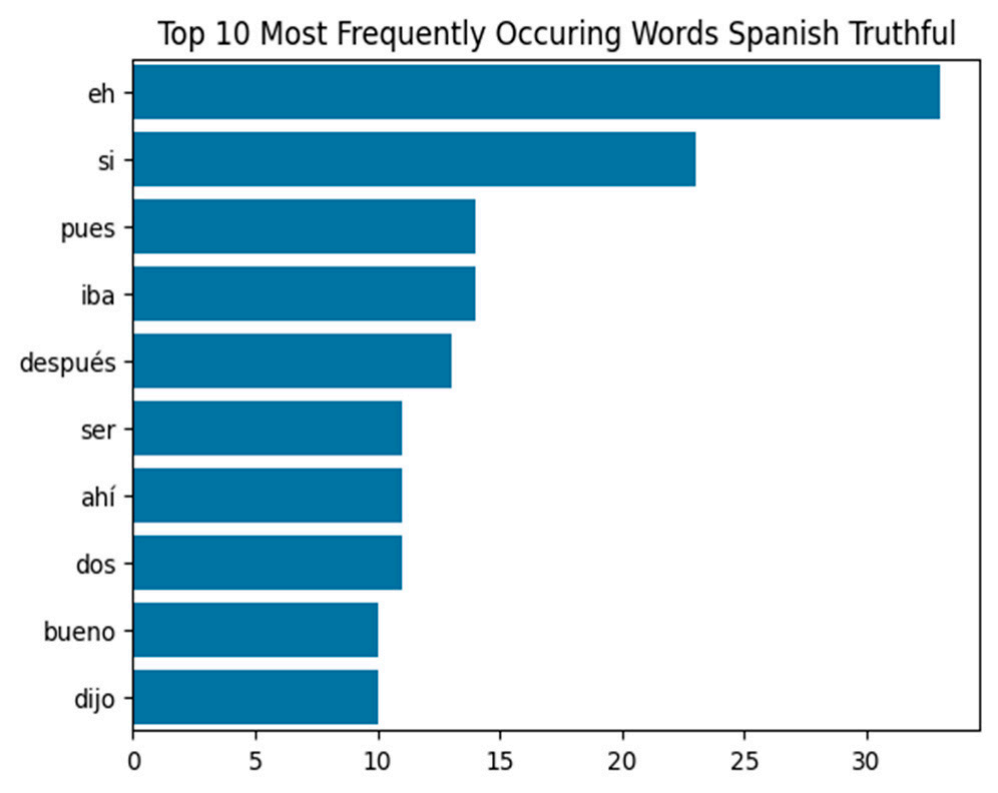
Top 10 Most Frequently Occurring Words in Spanish Data: Deceptive vs. Truthful, demonstrating lexical similarities and differences within the data. From most to least frequent Deceptive: eh (filler), si (‘yes’), pues (‘well’), hija (‘daughter’), bueno (‘good’), cosas (‘things’), dije (‘I said’), casa (‘house’), digo (‘I say’), sé (‘I know’). From most to least frequent Truthful: eh (filler), si (‘yes’), pues (‘well’), iba (‘was going’), después (‘after’), ser (‘to be’), ahí (‘there’), dos (‘two’), bueno (‘good’), dijo (‘said’).

Figure 8 shows the top words in deceptive and truthful transcripts. Though the first few words—‘eh’, ‘si’, and ‘pues’—appear in both categories, greater variation is seen in the rest of the top 10, helping the model differentiate between truth and deception. The Spanish visual model reached 92.86% accuracy—the lowest of the three languages. This lower performance can be attributed to the dataset’s small size and limited subject diversity. Still, like in English, the visual model alone may suffice when video is available. Notably, the Spanish audio model performed best overall, achieving 73.33% accuracy and 64.77% AUC. The cross-validation for audio justifies, this as Spanish remained high performing at a mean balanced accuracy of 86.96%. Unlike Tagalog speakers, who frequently code-switch, Spanish speakers in the dataset maintained consistent speech patterns and accents. This allowed the audio model to detect deception effectively. Given its strong performance and ease of audio recording in legal settings, audio could serve as the primary modality for deception detection in Spanish-speaking environments, reducing reliance on costly transcription or video analysis.

### 4.3. Tagalog Model: Individual Modality

The Tagalog textual model achieves 75% accuracy—outperforming the English model but falling short of the Spanish one. Like other languages, the Tagalog text data is processed using a Convolutional Neural Network (CNN). While CNN is good for the bag-of-words model, the relatively small Tagalog dataset could limit model performance. However, Tagalog’s unique syntax, structure, and conjugation—distinct from both English and Spanish—mitigate this limitation (Osoblivaia, 2023; Pascasio, 1961; Schachter & Reid, 2018). The model learns differently across languages, and performance is not solely dependent on dataset size. A key challenge in the Tagalog dataset is code-switching. Unlike Spanish subjects, who speak only Spanish, Tagalog speakers frequently mix English and Tagalog, increasing linguistic variability and making it harder for the model to identify consistent textual patterns. Even with cross-validation, which achieved 65.78% balanced accuracy, the Tagalog still performed better than the English dataset.

**Figure 9:**
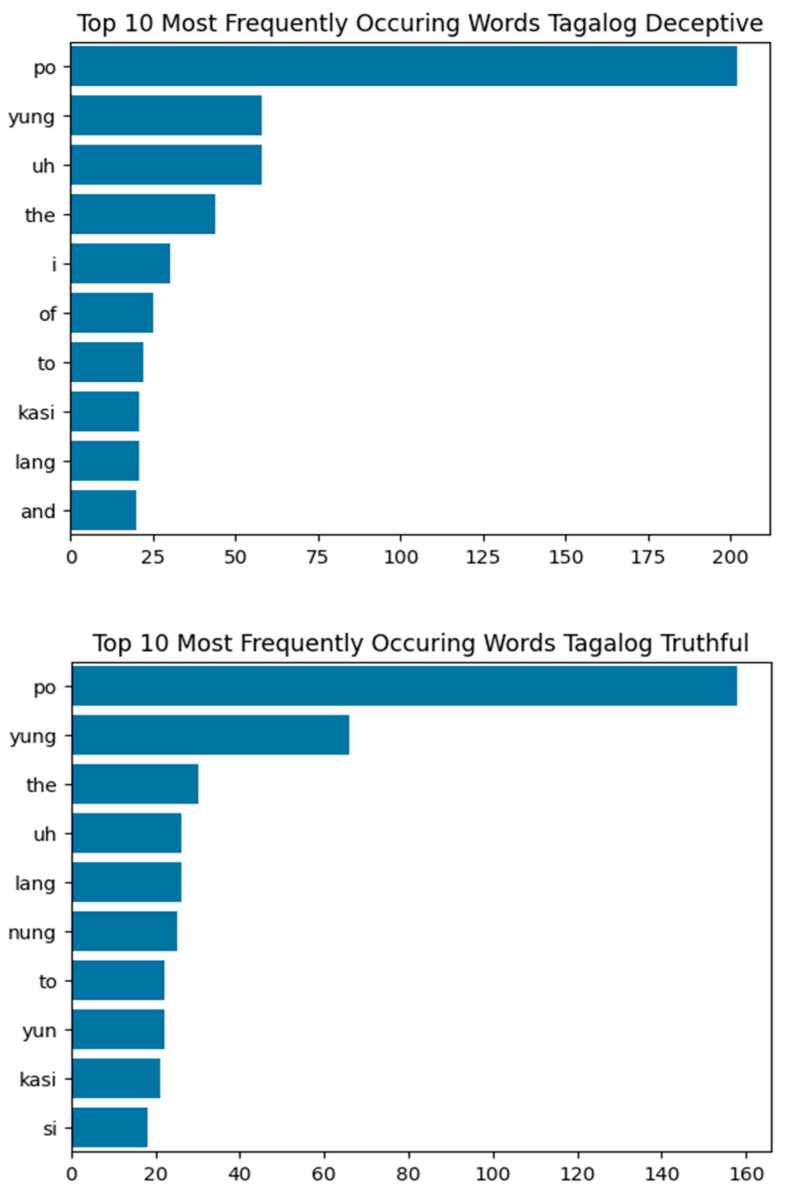
Top 10 Most Frequently Occurring Words in Tagalog Data: Deceptive vs. Truthful, demonstrating lexical similarities and differences within the data. From most to least frequent Deceptive: po (word used to make sentences formal), yung (‘that’), uh (filler), the, i, of, to, kasi (‘because’), lang (‘only’), and. From most to least frequent Truthful: po (‘sir/ma’am’), yung (‘that’), the, uh (filler), lang (‘only’), nung (‘when’), to, yun (‘that’), kasi (‘because’), si (personal topic marker that precedes names).

Figure 9 highlights the top words in deceptive and truthful categories. Though ‘po’, ‘yung’, ‘uh’, and ‘the’ appear in both, variations such as ‘nung’ and ‘yun’ in truthful statements indicate subtle linguistic differences that the model may exploit. The Tagalog visual model performs impressively, with 96.43% accuracy and 97.78% in cross-validation—surpassing Spanish and nearly matching English. The dataset’s homogeneity helped the model generalize visual cues effectively, suggesting that video alone could suffice for courtroom deception detection in Tagalog-speaking contexts. Unexpectedly, the Tagalog audio model was the weakest performer when trained in a normal split but had similar performance to English at 79.95% balanced accuracy after cross-validation. While pitch is a key feature, the standard deviation for all deceptive pitch values was 58.06%, and 59.88% for truthful—higher than truthful in English and Spanish (~40–45%) and lower than deceptive (~73–102%, respectively). This variability likely stems from inconsistent speech patterns and code-switching. Mixed-language sentences have different pitch contours and accents, complicating the model’s ability to distinguish deceptive behavior. Looking at the standard deviations of the other two languages in the deceptive and truthful categories shows a significant range between the two categories. The difference is clear. Unlike the other two languages, the audio model is not prepared for a bilingual speech pattern. Shen et al. also highlights how code switching has been difficult for speech recognition since many systems are trained on monolingual datasets. Shen et al. also stated that training on a bilingual or multilingual dataset should include preparation regarding how the acoustics and speech patterns are processed before training, suggesting that other researchers have combined models that were trained on monolingual datasets (Mustafa et al., 2022). Code switching in Tagalog was one of the biggest differences we saw compared to the other languages and a reason as to why we chose to analyze the language. We introduced a challenge to a model that may or may not be prepared for varying speech patterns of code switching per subject. Due to these inconsistencies, the Tagalog audio model struggles to find reliable patterns, reducing its standalone effectiveness in legal scenarios relying solely on audio.

**Figure 10:**
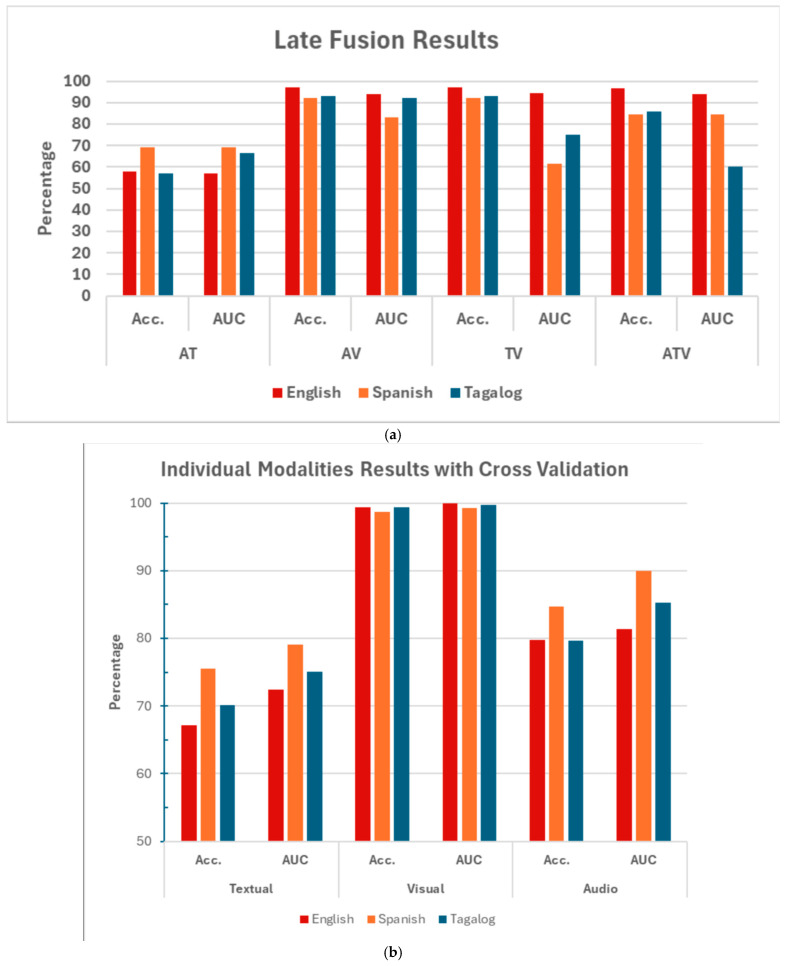
Accuracy and AUC for each single modality model by language, to compare the performance of individual modalities. (**a**) Accuracy and AUC from Normal Train/Test Split (75%/25%). (**b**) Mean Balanced Accuracy and AUC from most optimal *k*-fold Cross-validation.

Figure 10 summarizes the classification performance of each individual modality—text, audio, and visual—across English, Spanish, and Tagalog datasets. Overall, visual models consistently achieved the highest accuracy across all three languages, demonstrating their reliability in deception detection. Textual models showed strong performance in Spanish and Tagalog, while English lagged slightly, possibly due to greater subject variability. Audio models were the least consistent, with Spanish performing best and Tagalog the weakest in a normal split but achieving better performance evaluation during cross-validation, likely due to pitch variability and language mixing. These results highlight the visual modality as the most robust across languages, while audio performance is more sensitive to linguistic and acoustic diversity.

### 4.4. Multi-Modal Model by Late Fusion

Late Fusion refers to a model architecture that combines multiple modalities—Audio, Text, and Video—to improve deception detection. The main Late Fusion model, referred to as Audio–Text–Video (ATV), integrates all three modalities using a custom Weighted Average layer, where each modality is found to have a weight based on its relative contribution. In the initial stage of training, we chose to assign higher weights to the modality based on their individual performance and the other modality to have equal weights, and with the custom Weighted Average layer, these weights are adjusted based on the modal’s capability to lessen the loss function. We trained multiple times, decreasing the highest weight by one, until all weights are equal. For example, we saw that Visual had better metrics and assigned it a weight of 0.9, making the weights of Audio and Textual to be 0.05. Sub-models combining two modalities were also evaluated: Audio-Text (AT), Audio–Video (AV), and Text–Video (TV). These sub-models allow flexibility for environments where only limited types of data are available, such as courts lacking video recordings or transcripts. The weight assigned to these sub-models are similar to that of the main model. The Late Fusion architecture is particularly useful for addressing real-world constraints. Not all courtrooms, especially in smaller towns or under-resourced areas, can afford transcription services or video equipment. By comparing the performance of different modality combinations, we can identify effective and accessible deception detection setups tailored to such environments.

For the English language, the accuracy remained consistently high, with the ATV and TV models reaching near perfect performance across all weight combinations. This highlights that text and video worked especially well together. The AV model also performed well, though slightly below ATV and TV, while the AT model showed noticeably lower accuracy (mid-80s to low-90s), underscoring the importance of the visual modality. Even when audio and text were given greater weight, accuracy did not improve, emphasizing the importance of the visual modality in English deception detection. The main advantage of the consistent performance of Late Fusion on the English dataset is the large and diverse dataset. This lets us know that improving the dataset for the other languages is a must to achieve greater performance.

**Figure 11:**
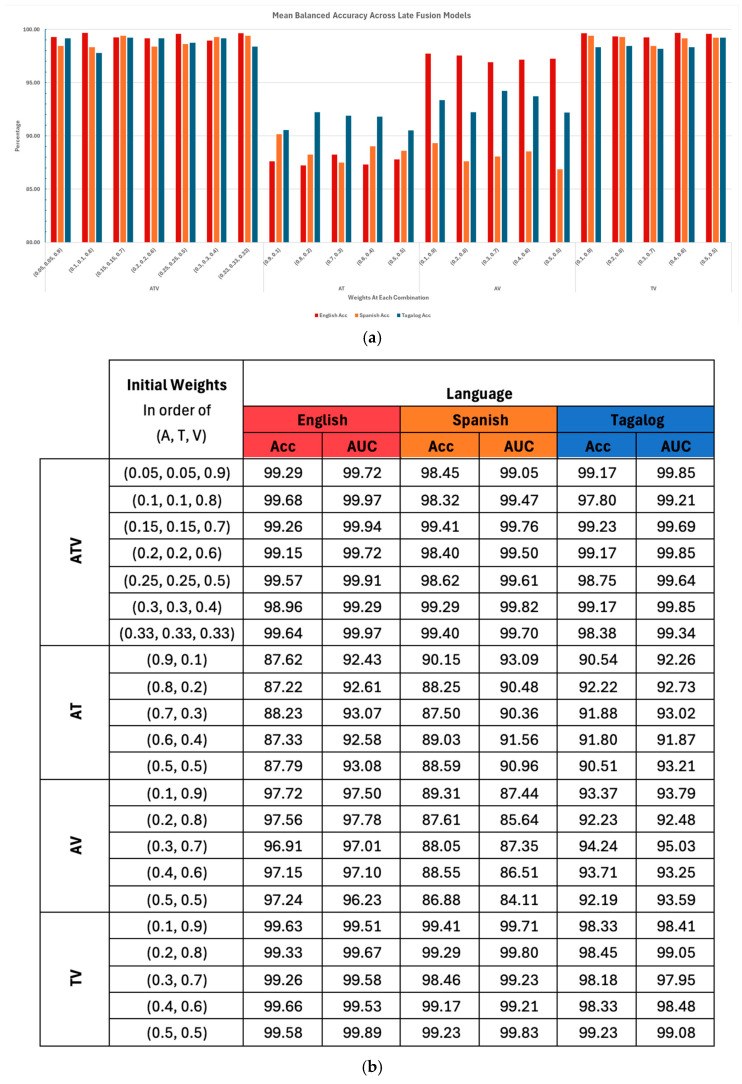
Balanced Accuracy and AUC for multi-modality late fusion models by language. (**a**) Clustered Column Chart displaying Mean Balanced Accuracy at different weight combinations. (**b**) All reported Mean Balanced Accuracy and AUC within weight combinations.

For the Spanish language, performance was more sensitive to weight distributions. The ATV model performed strongly overall, but the TV model often matched or outperformed it, suggesting that text added limited value. In Figure 11a, accuracy declined in configurations where Textual carried more weight (e.g., AT, TV), showing that the Spanish deception detection benefits most from the audio and visual modalities. This is surprising, since Spanish performed the best for the Textual category, but the results showed that it did not combine well with the other modalities compared to the other languages.

For Tagalog, the results emphasized the central role of the visual modality. The ATV and TV models consistently achieved the highest accuracy (up to ~99%), while the AT and AV models trailed behind. Notably, the AV performance improved when visual weights were higher but dropped with equal weighting, indicating that while audio may provide small gains, text contributed little, and models were most effective when visual input dominated. What is surprising, however, is that Tagalog was the best performing for the AT model. We noted earlier how Tagalog did not perform well individually due to code switching, but by combining the other modalities, detecting deception can be reliable. It is the only language to consistently hit greater than 90% accuracy for all weight distributions and sub-models. This is important considering that the purpose of the sub-models was to adapt to different environments where not all modalities were available.

Figure 11 illustrates how accuracy shifts across different weight combinations for ATV, AT, AV, and TV models in English, Spanish, and Tagalog. The ATV and TV models consistently reached the highest accuracies, particularly in English, where performance remained near 100% regardless of weight distribution. On the other hand, the AT sub-model showed a clear drop across all languages, while the AV model displayed more variability. These patterns visually confirm that the visual modality is the most reliable contributor, with its presence driving high overall accuracy across languages.

These findings reveal that while the visual modality consistently strengthens deception detection across languages, the utility of text and audio varies significantly. However, this consistency comes at a cost: computational power. We observed that preprocessing and analyzing video files is generally more computationally intensive than for the other two modalities thus having a longer processing time. Something to note, however, is that the English visual model and fusions with the visual model may be less reliable towards Asian subjects because of the fact that they are not as represented in the dataset compared to the other races. While adding the textual modality may lower the scores, using the bag-of-words system leads to the fastest processing time from the other modalities). Figure 11 summarizes the performance of Late Fusion models across English, Spanish, and Tagalog datasets using balanced accuracy and AUC scores. The English ATV model achieved the highest overall performance, benefiting from a larger and more balanced dataset. For Spanish, the Audio–Video (AV) model outperformed all others, showing that combining strong individual modalities yields the best results. In contrast, Tagalog’s ATV model underperformed due to weaker audio and text components, suggesting that the visual modality alone is most effective for deception detection in Tagalog-speaking courtrooms. Overall, visual data consistently contributed most to model performance across all languages. This analysis helps to inform how LegalEye can be adapted for courtrooms with varying resources and linguistic contexts.

## 5. Discussion and Conclusions

This paper presents LegalEye, a multimodal machine-learning model combining audio, visual, and textual analysis to detect deception in English, Spanish, and Tagalog courtroom settings. Using neural networks and late fusion, LegalEye achieves high accuracy across languages—97% for English, 85% for Spanish, and 86% for Tagalog—demonstrating its potential to improve fairness and reliability in legal proceedings. Machine learning can outperform human deception detection (Serra-Garcia & Gneezy, 2023), especially when used before individuals form opinions, countering confirmation bias. However, in trials, jurors hear testimony live, limiting real-time use of tools like LegalEye. Its greatest value lies in pre-trial interviews and depositions, providing analyses that inform legal strategy in both civil and criminal cases.

In the criminal justice system, bias often operates through implicit associations—subconscious connections influencing behavior despite conscious intentions to remain unbiased (Clemons, 2014). Most Americans harbor negative implicit biases toward Black Americans, linking Blackness and criminality in a reciprocal manner. Conversely, Asians are generally treated more leniently and receive lighter sentences than Black or Hispanic defendants, influenced partly by the “model minority” stereotype portraying Asians as hardworking and law-abiding (Johnson & Betsinger, 2009). These biases affect trial outcomes, and thus our dataset reflects implicit racial biases present in legal proceedings. Racial bias in AI systems often originates in the datasets used for training, which tend to overrepresent white males (Livingston, 2020). This bias can create feedback loops, such as inflated crime reporting in communities of color leading to increased police surveillance and further bias. Black men are disproportionately arrested and convicted, not because of higher crime rates, but due to systemic bias (Clemons, 2014). Asians are underrepresented in legal data, and research on their court treatment is scarce (Johnson & Betsinger, 2009). To mitigate bias, our dataset includes balanced numbers of White, Black, Hispanic, and Asian subjects, aiming for equal gender representation in truthful and deceptive categories. We matched racial ratios between truthful and deceptive groups for Whites, Blacks, and Hispanics but could not fully equalize Asian representation due to limited available data. This underrepresentation of Asians is important to note, as it may cause the model to learn weaker or less reliable patterns for this group. Consequently, outputs involving Asian subjects should be interpreted with caution.

This study has several limitations that warrant consideration. Video sourcing may introduce bias, as not all clips were recorded or broadcast prior to verdict announcements, and post-verdict media framing could have influenced their quality or context. In addition, the labeling of statements based on case verdicts, while consistent with established approaches in deception-detection research, does not ensure complete alignment between judicial outcomes and actual veracity, particularly in instances of wrongful convictions or acquittals. The editing and transcription process, although conducted according to standardized procedures and designed to minimize subjectivity, may still have introduced minor inconsistencies. Taken together, these factors caution against broad generalization of the results beyond the specific dataset analyzed and underscore the need for external validation using independent and more diverse trial recordings to substantiate the robustness of the findings.

Rather than relying on fixed behavioral cues that have historically shown limited reliability in deception research, our models identify deception through aggregated multimodal patterns. Specifically, the integration of facial action units and emotion vectors, vocal features such as pitch variation and MFCCs, and linguistic markers related to word frequency and structure allows the model to capture complex interactions that may not be discernible through any single modality. This approach emphasizes that deception detection in LegalEye does not depend on universal behavioral cues, but instead on statistical regularities that emerge across modalities, thereby addressing longstanding concerns about the inconsistency of traditional cue-based approaches.

Deception detection plays a role in a variety of legal settings, both in and out of the courtroom. In a survey of employment, contract, and tort cases, settlement rates were found to be around 70% to 80% across jurisdictions, making up a large proportion of cases (Eisenberg & Lanvers, 2009). In these cases, lawyers and clients analyze available evidence and subject interviews to assess their position and the strength of their case. Analytical tools such as LegalEye that are capable of predicting how a subject’s testimony will come across in court—either as truthful or deceptive—can improve legal counsel’s ability to advise their clients and influence legal negotiations. In the criminal sphere, negotiations known as pleas make up “almost 98 percent of federal convictions and 95 percent of state convictions in the United States,” according to the American Bar Association (Dervan, 2024). Nonetheless, it is important to clarify that, even in pre-trial settings, LegalEye can carry risks if users rely too strongly on the model scores. It is possible that LegalEye’s predictions may shape decisions or reinforce existing biases, so to mitigate these concerns, future implementations of the model will integrate Explainable AI techniques such as SHAP values. SHAP values will reveal which specific features most influenced the model’s prediction, such as changes in pitch or eye movements, allowing users to better understand and analyze deception indicators. This transparency will enable users to assess and discern their trust in the model and its output, reducing blind reliance on the model’s output.

LegalEye currently supports English, Spanish, and Tagalog legal settings. These languages were chosen due to the availability of training data—curated from online courtroom videos and legal interviews—and access to scribes for manual transcription. However, in the future, we would like to add support for additional languages if quality data and means of employing additional scribes are available. Moreover, to increase transcription accuracy, we plan on assigning multiple scribes to each language. Although automated transcription methods exist, the manual process prevents an additional AI model from introducing bias to the transcriptions.

We also believe that expanding our existing datasets with additional videos will improve LegalEye’s deception detection capabilities, as small datasets can hinder model training and performance. To this end, future work should emphasize the collection of additional videos as new trial samples become available online. Additionally, the visual modality is currently the most impactful sub model, playing a key role in the late fusion model’s performance. Despite this, some individuals and even courtrooms may lack the equipment to record videos, while transcripts and audio recordings may be possible instead. For this reason, future work should focus on improving the performance of the textual and audio models, allowing LegalEye to be effective in additional contexts.

## Figures and Tables

**Table 1:** Racial composition of our English dataset.

Verdict	Black	White	Hispanic	Asian	Total
Deceptive	40	40	40	5	125
Truthful	41	40	41	8	130
Total	81	80	81	13	255

**Table 2:** Ground truth distribution for each language dataset.

Language	Deceptive	Truthful
English	125	130
Spanish	40	69
Tagalog	60	52
Total	225	251

## Data Availability

The training videos are sourced from publicly available YouTube content and have been organized on this page (accessed on 1 December 2025): https://docs.google.com/spreadsheets/d/1Q09nuzN3zPfJ7rrWQUBCS5ePL4Qcc2czVgd3cwhmoCI/edit?usp=sharing.

## Abbreviations

The following abbreviations are used in this manuscript:

AI	Artificial Intelligence
ML	Machine Learning
RNN	Recurrent Neural Network
CNN	Convolutional Neural Network
MFCC	Mel-frequency Cepstral Coefficient
AUC	Area Under the Receiver Operating Characteristic Curve
ATV	Audio–Text–Video
AT	Audio–Text
AV	Audio–Video
TV	Text–Video
